# Obsessive-compulsive disorder of vowels (rare type)

**DOI:** 10.4103/0019-5545.58306

**Published:** 2009

**Authors:** V. Anandprakash Ghorpade

**Affiliations:** Consultant Psychiatrist, 1103, 24^th^ Main, J.P. Nagar, I Phase, Bangalore, India. E-mail: ghorpade.vap@gmail.com

Sir,

Patients presenting with unique and rare symptoms are the best teachers of clinicians, encouraging and enabling them to become wise with passage of time. This patient presented with an obsession of VOWELS. Literature survey failed to show such documented cases, hence brief details of this unusual and rare case is presented here.

A 40-year-old married male, a Christian, whose mother tongue was English, and who working as an instructor in a defense organization, presented with complaints of an irrational fixed idea of searching for vowels and consonants in the text material which he came across, resulting in intense anxiety, breathing difficulty, and confused thinking since eight years.

As a child he had an extraordinary memory (after reading a book once, he could reproduce it word by word). He used to be a topper all the time, till the tenth standard, which made his relatives to have high expectations from him. This made him preoccupied with studies and ranks, almost all the time, with no inclination for relaxation. When he joined the eleventh standard in a different school he had to compete with many toppers who had joined that school, unlike the old school where he was the only topper. Despite this he was within the sixth or seventh rank, which disappointed him, but he continued to work hard. With this changed situation, his ambition was to be at least within the third position.

In the year 1983, when he was 17 years old, just before attending his eleventh standard exam in English, he tried to recall what he had read and to his surprise he found his mind was blank, resulting in fear. Subsequently this fear spread to other exams also. Despite this, he could write competitive exams and come out with flying colors. One year later he developed viral fever following which he realized that his irrational fear of not being able to recall and read relapsed. With minor fluctuations in his distressful symptoms he graduated as an Engineer.

He was preparing for a prestigious competitive exam during which he had a vague feeling in his mind that he should not fail. With this background, in the month of January 1998, when he was working as an instructor in a defense establishment, he was exposed to different names of squadrons with alphabets.

While talking or reading he developed an urge in his mind to look for the first vowel, the sight of which used to make him anxious, which was characterized by shortness of breath, anxiety, and confused thinking, which used to last for a few minutes to few hours and remit spontaneously. This used to appear during his exams and the rest of the time he used to be apparently normal.

From 1999 to 2000 he did not have any problem with the vowels. His achievement of clearing the competitive exam brought him a lot of appreciation.

During the period 2000 to 2003 he had mild relapses, which he could control, and his work continued without any interruptions.

In the month of January 2003, he was transferred to another regiment where instead of vowels, his anxiety used to get triggered by whole words with a vowel, for example, Danger; this used to happen both while reading and talking. At this stage he consulted a psychiatrist who diagnosed him with Obsessive-Compulsive Disorder (OCD) and prescribed selective serotonin reuptake inhibitors (SSRI). He took the treatment irregularly and also did not visit the psychiatrist regularly. SSRI made him calm, but the primary problem remained unchanged. In April 2006, the vowel A was replaced by the next vowel I. Then the other vowels, consonants, and specific numbers used to provoke anxiety. The numbers he mentioned were 6 and 9, the basis for these numbers becoming anxiogenic was the presence of vowels in the words six and nine. This affected his work connected with calculations. During April 2007, he started looking for combinations of vowels such as A and E in words and sentences.

Physical examination revealed no abnormalities. Mental status examination revealed that he was well-dressed, anxious, rigid while talking, with no evidence of any psychosis, cognitive functions were intact, no other obsessions, compulsions or phobias were elicited, with good judgment and insight.

He is a voracious reader, a social drinker and non-smoker, physically hale and healthy. His attitude was one of a perfectionist, anxious, God fearing, not very religious, liked to be honest, with a strong desire to excel in all his endeavors.

He described his father as being outspoken and an extrovert, whereas, his mother was shy and anxious. He hailed from a family where there were no heritable diseases.

As he expressed his desire to be treated without drugs, behavioral psychotherapeutic techniques were used to treat him, about four sessions, which yielded encouraging results.

Summarizing the case: Middle-aged male with features of obsessive traits, with high level of confidence and positive encouragement from his relatives, developed vague fear, which over the course of time focussed on vowels, slowly spreading to other vowels, words and so on, with a deep unexpressed wish to go back to his sweet life before the age of 16.

Highlights of this case are preoccupation with vowels at times, which evoked a state of anxiety in the patient, with a tendency to avoid it. This recurring intrusive idea, which is recognized as irrational by the patient, resulting in his discomfort, and him anxiously looking for it, is suggestive of obsessive disorder.

There were no compulsions associated with it. A diagnosis[1] of OCD of vowels was made. Literature survey did not yield any results in relation to this unusual obsessive content.

On account of his inability to attend the psychotherapeutic sessions further, he agreed to keep in touch with the therapist through e-mail.
